# A Comparative Study of Diffusion Fiber Reconstruction Models for Pyramidal Tract Branches

**DOI:** 10.3389/fnins.2021.777377

**Published:** 2021-12-09

**Authors:** Xinjun Suo, Lining Guo, Dianxun Fu, Hao Ding, Yihong Li, Wen Qin

**Affiliations:** ^1^Department of Radiology, Tianjin Medical University General Hospital, Tianjin, China; ^2^Tianjin Key Lab of Functional Imaging, Tianjin Medical University General Hospital, Tianjin, China; ^3^School of Medical Imaging, Tianjin Medical University, Tianjin, China

**Keywords:** diffusion tensor imaging, diffusion spectral imaging, generalized Q-space sampling imaging, Q-ball imaging, pyramidal-tracts branches

## Abstract

Currently, comparative studies evaluating the quantification accuracy of pyramidal tracts (PT) and PT branches that were tracked based on four mainstream diffusion models are deficient. The present study aims to evaluate four mainstream models using the high-quality Human Connectome Project (HCP) dataset. Diffusion tensor imaging (DTI), diffusion spectral imaging (DSI), generalized Q-space sampling imaging (GQI), and Q-ball imaging (QBI) were used to construct the PT and PT branches in 50 healthy volunteers from the HCP. False and true PT fibers were identified based on anatomic information. One-way repeated measure analysis of variance and *post hoc* paired-sample *t*-test were performed to identify the best PT and PT branch quantification model. The number, percentage, and density of true fibers of PT obtained based on GQI and QBI were significantly larger than those based on DTI and DSI (all *p* < 0.0005, Bonferroni corrected), whereas false fibers yielded the opposite results (all *p* < 0.0005, Bonferroni corrected). More trunk branches (PT_trunk_) were present in the four diffusion models compared with the upper limb (PT_Ulimb_), lower limb (PT_Llimb_), and cranial (PT_cranial_) branches. In addition, significantly more true fibers were obtained in PT_trunk_, PT_Ulimb_, and PT_Llimb_ based on the GQI and QBI compared with DTI and DSI (all *p* < 0.0005, Bonferroni corrected). Finally, GQI-based group probabilistic maps showed that the four PT branches exhibited relatively unique spatial distributions. Therefore, the GQI and QBI represent better diffusion models for the PT and PT branches. The group probabilistic maps of PT branches have been shared with the public to facilitate more precise studies on the plasticity of and the damage to the motor pathway.

## Introduction

White matter fiber tracking based on the diffusion tensor imaging (DTI) model has been widely available and is often used in clinical practice and clinical neuroscience to examine the brain’s white matter (WM) microstructures (Mori and Zhang, 2006; Chen et al., 2011; Thomason and Thompson, 2011; Auriat et al., 2015; Chaudhary et al., 2015; Lucas-Jiménez et al., 2015; Lope-Piedrafita, 2018; Tae et al., 2018). The DTI model is used primarily for the diagnosis, treatment, and prognosis assessment of brain diseases because it is a non-invasive medical imaging tool that displays WM fibers *in vivo* (Robinson et al., 2010; Kim et al., 2015; Song et al., 2015; Atkinson-Clement et al., 2017). The DTI model has also provided considerable insight into our understanding of functional connectivity based on functional magnetic resonance imaging (fMRI), revealing subtle alterations in the WM microstructure between different functional networks (Cunningham et al., 2017; Li et al., 2018; Larabi et al., 2020).

However, the DTI model can only characterize the main diffuse direction in a voxel and cannot distinguish the crossed fibers within the voxel due to the limitation of the number of diffusion-sensitive magnetic field gradients (Oouchi et al., 2007; Chung et al., 2013; Young et al., 2017). For example, the DTI model displays relatively straight fibers of the pyramidal tract (PT) better than cross-fibers (Oouchi et al., 2007). In recent years, many researchers have proposed a variety of improved diffusion direction quantitative models to achieve the tracking of crossed fibers and have more realistically revealed the connection of brain networks, including diffusion spectrum imaging (DSI) (Yeh and Verstynen, 2016), q-space spherical imaging (Q-ball imaging, QBI) (Descoteaux et al., 2007; Aganj et al., 2009), and generalized q-sampling imaging (GQI) (Yeh et al., 2010).

The PT comprises the corticospinal tract (CST), which is in charge of monitoring movements of the limbs and trunk, and the corticobulbar tract (CBT), which directs the face, head, and neck. The PT is a major neuronal pathway emitted from the primary motor cortex (M1) to mediate voluntary movements. The brain region of M1 spans approximately half of the cerebral cortex, and its distribution from the inner-upper to the outer-lower region is consistent with allocation areas ranging from the foot to the head and corresponding to lower limb (PT_Llimb_), upper limb (PT_Ulimb_), trunk branches (PT_*trunk*_), and cranial (PT_cranial_) branches (Fan et al., 2016). Anatomically, the WM fiber bundle of the PT is distributed in a fan shape, collected in the posterior limb of the inner capsule, and concentrated downward through the ipsilateral cerebral peduncle and pons (Canedo, 1997). A portion of the fiber bundle is activated in the deep brain nucleus of the brainstem to allocate the skeletal muscle of the contralateral head, and most of the fiber bundle crosses to the opposite side in the lower part of the medulla (Fulton and Sheehan, 1935; Davidoff, 1990) and is finally activated in the anterior horn of the spinal cord to allocate skeletal muscle of the contralateral limbs (Davidoff, 1990; Canedo, 1997; Javed and Lui, 2019). PT injury is typically accompanied by paralysis, and the specific paralyzed parts of the body correspond to different branches of PT (Jang, 2009; Li et al., 2020; Pennati et al., 2020). Therefore, the map of PT branches is of great significance to clinical diagnosis and prognosis guidance. However, most of the previous studies on the WM fibers of the PT were exclusively based on the DTI diffusion model or one of the other three emerging diffusion models (Oouchi et al., 2007; Wang et al., 2016; Schilling et al., 2017; Bao et al., 2019; Suo et al., 2021), and the focus of these studies was primarily limited to the whole fibers of the PT rather than each PT branch. Meanwhile, the anatomical travel of PT is concentrated and the variation is small in comparison with other fiber bundles, which can be used as a typical demonstration reference for evaluating different models. At present, there is no PT branch map based on the definition of functional dominance area; one of the purposes of this research is to provide a fine PT branch as a tool for the research and clinical application.

Here, we aim to quantitatively evaluate the advantages and disadvantages of several mainstream diffusion mathematical models (DTI, DSI, QBI, and GQI) in displaying intracranial PT and PT branches based on the Human Connectome Project (HCP) dataset, providing an objective reference for the selection of diffusion models in clinical or scientific research. Moreover, we shared groupwise probabilistic maps of the PT branches based on the best diffusion model for the public to more precisely study the plasticity and the damage of the motor pathway.

## Materials and Methods

### Participants

The HCP is a program funded by the National Institutes of Health to map neural pathways related to brain function and behavior. The program provides scientific researchers with ultrahigh-quality multimodal brain magnetic resonance data and shares its acquisition and analytical criteria. The most sophisticated scanning scheme for the human brain is applied in diffusion imaging, making it suitable for various traditional or complex diffusion mathematical models (Glasser et al., 2016). Fifty healthy adults (age: 22–35 years old, 25 males and 25 females) from the Q1 stage of HCP were randomly collected in our study. We included healthy young adults that could accept the telephone diagnostic interview and provide the valid informed consent. The exclusion criteria included (1) significant history of psychiatric disorder, substance abuse, or neurological or cardiovascular disease; (2) two or more seizures after 5 years of age or a diagnosis of epilepsy; (3) any genetic disorder, such as cystic fibrosis or sickle cell disease; (4) multiple sclerosis, cerebral palsy, brain tumor, or stroke; (5) head injuries; (6) premature birth; (7) currently on chemotherapy or immunomodulatory agents or history of radiation or chemotherapy that could affect the brain; (8) thyroid hormone treatment in the past month; (9) treatment for diabetes in the past month; (10) use of daily prescription medications for migraines in the past month; (11) a score of 25 or less on the Folstein Mini Mental State Exam on visit Day 1; (12) moderate or severe claustrophobia; (13) pregnancy; and (14) unsafe metal in the body. More details of recruitment information for HCP subjects are provided at the website https://www.humanconnectome.org.

### Structural Magnetic Resonance Imaging and Diffusion Magnetic Resonance Imaging Data

MRI data from the HCP dataset were scanned on a 3.0-T WU-Minn-Ox HCP scanner with very strong magnetic field gradients (100 mT/m) along with state-of-art diffusion magnetic resonance imaging (dMRI) pulse sequences; the detailed parameters of all MR data are available at the official website.^1^ The magnetization prepared for rapid acquisition gradient-echo (MP-RAGE) sequence was used to obtain 3D T1-weighted structural MRI (sMRI) data with the following parameters: repetition time (TR)/echo time (TE) = 2,400/2.14 ms, flip angle = 8°, field of view (FOV) = 224 mm × 224 mm, matrix = 320 × 320, and slice thickness = 0.7 mm, resulting in a 0.7-mm isotropic voxel.

The multiband, single-shot, spin-echo echo-planar imaging (MB-SS-SE-EPI) sequence was used to obtain dMRI (Glasser et al., 2016). The main parameters used in dMRI were as follows: TR/TE = 5,520/89.5 ms, flip angle = 78°, multiband factor = 3, FOV = 210 mm × 180 mm, matrix = 168 × 144, slice thickness = 1.25 mm, 111 slices, and 1.25 × 1.25 × 1.25 mm^3^ isotropic voxel. dMRI consisted of six runs, which corresponded to three shells of b = 1,000, 2,000, and 3,000 s/mm^2^, and each table was repeated with right-to-left and left-to-right phase encoding directions. There are 90 diffusion-weighted directions plus six b0 acquisitions interspersed throughout each run. The diffusion data used in our study were preprocessed according to the minimal preprocessing pipelines provided by the HCP website. FSL6.0 software^2^ was used for data preprocessing. The minimal preprocessing pipelines consist of intensity normalization, distortion correction (FSL topup toolbox), image motion correction (FSL eddy toolbox), gradient non-reality correction, b0-to-structure linear coregistration, and brain extraction (FSL bet) (Glasser et al., 2013).

### Four Diffusion Reconstruction Models

We performed reconstruction to acquire the orientation distribution function of each voxel in the native space using four diffusion models based on DSI Studio 2017.^3^ DSI Studio 2017 can construct various mainstream diffusion models and perform real-time visualization for fiber tracking (Yeh et al., 2016).

The DTI diffusion model assumes that the diffusion of water molecules in all directions follows a single exponential decay law. Only one dispersion exists in the main direction. The first-order tensor model is used to simulate the direction distribution of the diffusion coefficient, and the eigenvalue and eigenvector are obtained by decomposition. The largest eigenvector is the main direction of the fiber in the eigenvalue voxels. However, the results of the model-based approach are always limited by the model. The diffusion model does not often meet the assumptions, and overfitting problems may exist in complex models. The DTI model in this study used a standard linear least square method to solve the tensor matrix proposed by Basser et al. (1994). The parameters used in the DTI model are recommended by DSI Studio 2017.

The DSI diffusion model is a model-free method that estimates the empirical distribution of water diffusion and makes no assumptions about the distribution. The DSI model calculates the probability distribution function (PDF) and the orientation distribution function (ODF) of water diffusion using Fourier transformation and numerical integration (Wedeen et al., 2005). First, the 3D Fourier transform is performed on the q-space data to obtain the PDF; here, multiple directions of *b*-values are needed because the Fourier transform requires a specific mesh diffusion sampling scheme. Next, integrating several discrete points in each sampling direction in the PDF is used to estimate the ODF. Given the fact that the ODF contains multiple diffuse principal directions, it is possible to characterize various intersecting fibers within a voxel. The DSI model used in this study was proposed by Wedeen et al. (2005). The Hanning filter factor was 0.17. The other parameters used in the DSI model are recommended by DSI Studio 2017.

The QBI diffusion model, which is one of the model-free methods, can use Funk–Radon transforms or spherical harmonic functions to directly calculate the ODF without a PDF. The QBI diffusion model in our study was based on spherical harmonic transformation according to the research of Descoteaux et al. (2007). The parameters used in this study included QBI regularization = 0.006 and decomposition fraction = 0.05.

By quantifying the diffuse density of water in different directions, the GQI provides a quantitative relationship between the diffuse signal and the spin distribution function (SDF), which can be applied to any diffuse sampling scheme (Yeh et al., 2010). Studies have shown that SDF is more sensitive and specific to the characteristics and pathology of the brain WM. GQI can calculate SDF based on various diffusion datasets, including DSI datasets, HARDI, multilayer shells, and joint DTI datasets. The GQI diffusion model provides an analysis relationship for SDF calculation, and the reconstruction only requires simple matrix multiplication (Yeh et al., 2010). The parameters used in this study included diffusion sampling length ratio = 1.25 and decomposition fraction = 0.05.

In DTI studies, fractional anisotropy (FA) is always used to measure the coherence of diffusion in one particular direction. It should be noted that the FA generated with DSI, QBI, or GQI reconstruction in DSI Studio is an index called the quantitative index (QA) (Yeh et al., 2010), and the index used in our research is normalized quantitative anisotropy (nQA: scaled from QA so that the maximum nQA of the subject is one).

### The Selection of Brain Masks for Pyramidal Tracts

**FIGURE 1:**
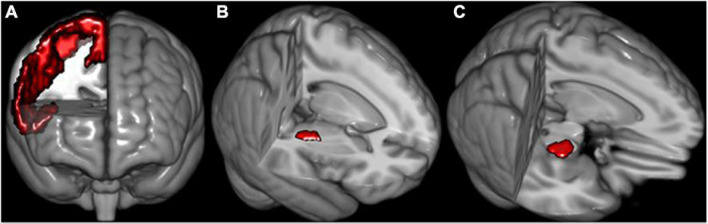
The schematic of seeds used for fiber reconstruction. **(A)** Right primary motor cortex (M1). **(B)** Right internal capsule occipital. **(C)** Right anterior cerebral peduncle. The definition of seeds of the left hemisphere is similar to the right side.

During fiber tracing of right PT, right M1 is the seed, the right internal capsule occipital was used as waypoint, and the anterior cerebral peduncle was used as avoidance; during fiber tracing of left PT, left M1 is the seed, the left internal capsule occipital was used as waypoint, and the anterior cerebral peduncle was used as avoidance (Figures 1A–C). The Brainnetome atlas consists of 210 cortical and 36 subcortical subregions, which were parcellated based on the anatomical connectivity patterns between voxels (Fan et al., 2016). Therefore, this atlas is preferable for connection-based analysis.^4^ We further parcellated M1 into four subregions: paracentral lobule (PCL)_R_2_2 (PT_Llimb_), precentral gyrus (PrG)_R_6_3 (PT_Ulimb_), PrG_R_6_4 (PT_trunk_), PrG_R_6_1, and PrG_R_6_5 (PT_cranial_). Of note, the brain regions used in the fiber tracking were inversely transformed into the native diffusion space of each subject. Here, we conducted the two preceding registration steps (i.e., b0-to-T1 and T1-to-MNI) and inverse transformation. First, the FLIRT function was used to affinely co-register the b0 images to the sMRI space (b0-to-T1). Next, the FNIRT function was used to non-linearly normalize the sMRI into MNI space (T1-to-MNI). Then, concatenation of the two preceding registration steps was used to calculate the b0-to-MNI deformation parameters, i.e., b0-to-T1 and T1-to-MNI (applywarp function). Finally, we applied the deformation parameters on the brain regions to inversely warp them into the subjects’ native diffusion space for fiber tracking and quantification. The brain regions were composed of the internal capsule occipital region, the anterior cerebral peduncle, the M1 region, and its parcellation subregions. All the steps of fiber tracking were performed in the individual diffusion space. We used FSL6.0 software (see text footnote 2) to accomplish the above preprocessing.

### The Tracking Parameters for Pyramidal Tracts and Its Branches

Deterministic fiber tracking was performed to acquire PT and PT branches. The angle threshold we chose was 60°, and the step size was 0.625 mm (1/2 voxel size) (Wang et al., 2013). The criterion for selecting the anisotropic score (FA or nQA) was that the cerebrospinal fluid of the bilateral ventricles was not covered in the range of fiber tracking, and all WM was covered. The tracked fibers were smoothed by averaging the propagation direction with a 20% average of the previous direction (Fernandez-Miranda et al., 2015). We removed fibers with a length that was outside the range of 30–300 mm (Ghulam-Jelani et al., 2021). The tracking termination condition was the number of sampling points of 10^7^ (Zdanovskis et al., 2021). Finally, the true fibers and false fibers of the PT in each subject were distinguished for subsequent visual presentation and quantification analysis according to anatomical knowledge and the functions for processing fibers provided by DSI Studio 2017 (see text footnote 3).

### The Definition of “True” and “False” Fibers in Fiber Tracking

Two radiologists participated in the description of true and false fibers based on anatomical knowledge. The traced fibers are divided into several parts. After carefully confirming the true and false fibers in each part, the true and false fibers are combined separately. False fibers mainly refer to abnormal anatomical traveling, such as (1) fibers that reached the contralateral cerebral hemisphere through the corpus callosum and (2) fibers that reached the contralateral cerebral hemisphere through the pons.

### The Groupwise Probabilistic Maps

The fiber bundles of each PT branch based on the GQI diffusion model for each subject were spatially normalized into the MNI space *via* the b0-to-MNI deformation parameters. Then, each PT branch’s groupwise fiber probability map was generated by averaging the normalized individual fibers.

### Statistical Analysis

In our study, four different diffusion models (i.e., DTI, GQI, QBI, and DSI) were compared using eight measures, including (1) the number of true fibers, (2) the number of false fibers, (3) the percentage of true fibers among the total number of fibers, (4) the percentage of false fibers among the total number of fibers, (5) the number of true fibers in PT_*cranial*_, (6) the number of true fibers in PT_Ulimb_, (7) the number of true fibers of PT_*trunk*_, and (8) the number of true fibers in PT_*Llimb*_. Thus, 16 comparisons were performed for the left and right sides of these measures. Specifically, we first performed one-way repeated measure analysis of variance (ANOVA) for each measure, and the significance level was set at Bonferroni corrected *p* < 0.05/16 = 0.003. Then, we performed *post hoc* analysis by conducting the paired-sample *t*-test between any two diffusion models in the measures that exhibited a significant difference in ANOVA, and the significance level of *post hoc* analysis was set at Bonferroni corrected *p* < 0.05/16/6 = 0.0005 (0.05/the total number of ANOVA models/the total number of paired-sample *t*-tests). It is worth noting that in order to eliminate the interference of the volume of the region of interest (ROI), we also supplied the evaluation of fiber density (number of fibers divided by the volume of the seed).

## Results

### Pyramidal Tracts Fibers Tracked Based on Four Diffusion Models

**FIGURE 2:**
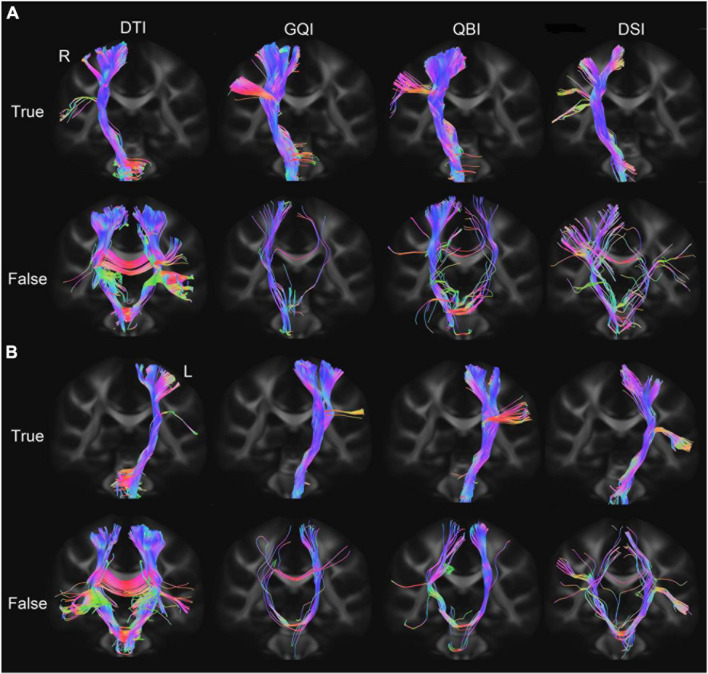
PT fibers tracked based on four diffusion models. **(A)** The true and false fibers of the right PT. **(B)** The true and false fibers of the left PT. DTI, diffusion tensor imaging; DSI, diffusion spectral imaging; GQI, generalized Q-space sampling imaging; L, left; PT, pyramid tract; QBI, Q-ball imaging; R, right.

The bilateral PT of 50 subjects was tracked based on four diffusion models (Figure 2). The true fibers mainly terminated in the upper part of M1. The GQI and QBI better displayed the full profile of PT compared with the other diffusion models. The number of false fibers tracked based on the DTI diffusion model was the highest among the four models. The travel path of false fibers mainly included (1) fibers that reached the contralateral cerebral hemisphere through the corpus callosum and (2) fibers that reached the contralateral cerebral hemisphere through the pons.

### Comparison of the Quantification of True Fibers Based on Four Diffusion Models

**FIGURE 3:**
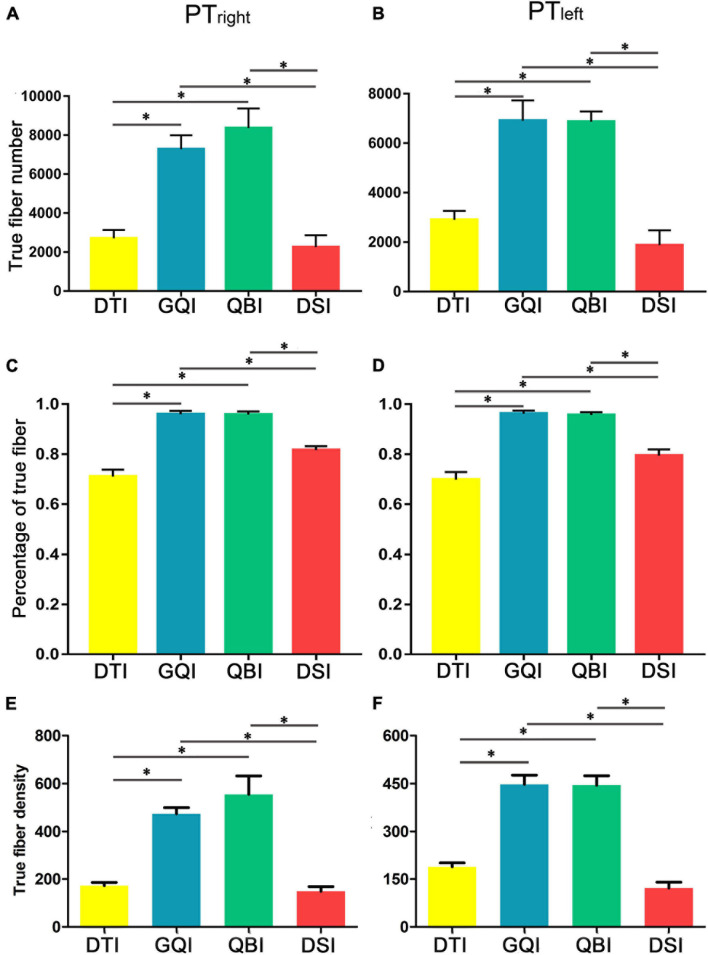
Comparison of the true fiber number and density tracked based on four diffusion models. **(A)** The true fiber number of the right PT. **(B)** The true fiber number of the left PT. **(C)** The percentage of true fibers of the right PT. **(D)** The percentage of true fibers of the left PT. **(E)** The true fiber density of the right PT. **(F)** The true fiber density of the left PT. DTI, diffusion tensor imaging; DSI, diffusion spectral imaging; GQI, generalized Q-space sampling imaging; L, left; PT, pyramid tract; QBI, Q-ball imaging; R, right. * represents *p* < 0.0005 (0.05/96, Bonferroni corrected). The error bar represents standard errors.

Regarding the number of true fibers of bilateral PT (Figures 3A,B), ANOVA revealed statistically significant differences among the four diffusion models (right PT: *F* = 33.01, *p* = 6.81 × 10^–16^; left PT: *F* = 102.56, *p* = 3.83 × 10^–26^; Supplementary Table 1). Meanwhile, *post hoc* analysis was performed between any two diffusion models (Supplementary Table 2). The number of true fibers tracked based on the GQI diffusion model was significantly greater than those tracked with DTI (right PT: *p* = 8.38 × 10^–19^; left PT: *p* = 6.67 × 10^–14^) and DSI (right PT: *p* = 1.05 × 10^–14^; left PT: *p* = 3.47 × 10^–16^), and the number of true fibers tracked based on the QBI diffusion model was significantly higher than those tracked with DTI (right PT: *p* = 1.09 × 10^–6^; left PT: *p* = 4.78 × 10^–14^) and DSI (right PT: *p* = 3.66 × 10^–7^; left PT: *p* = 4.92 × 10^–16^). No significant difference was discovered between the GQI and QBI (right PT: *p* = 0.28; left PT: *p* = 0.82) or between the DTI and DSI (right PT: *p* = 0.22; left PT: *p* = 5.99 × 10^–3^). Regarding the right PT, the rankings of the number of true fibers obtained by four diffusion models from greatest to least were as follows: QBI, GQI, DTI, and DSI (Figure 3A and Supplementary Table 1). For the left PT, the rankings of the number of true fibers obtained by the four diffusion models from greatest to least were as follows: GQI, QBI, DTI, and DSI (Figure 3B and Supplementary Table 1). The results of the percentage of true fibers (the number of true fibers divided by the total number of fibers) were similar to the number of true fibers (Figures 3C,D and Supplementary Tables 1, 2); one-way repeated-measure ANOVA revealed statistically significant differences in the four diffusion models (right PT: *F* = 49.70, *p* = 6.05 × 10^–14^; left PT: *F* = 45.38, *p* = 3.11 × 10^–14^; Supplementary Table 1). The percentage of true fibers tracked based on the GQI diffusion model was significantly greater than those of DTI (right PT: *p* = 5.74 × 10^–10^; left PT: *p* = 1.97 × 10^–11^) and DSI (right PT: *p* = 3.97 × 10^–9^; left PT: *p* = 1.77 × 10^–8^), and the percentage of true fibers tracked based on the QBI diffusion model was significantly greater than those of the DTI (right PT: *p* = 1.79 × 10^–11^; left PT: *p* = 1.16 × 10^–11^) and DSI models (right PT = 4.36 × 45 × 10^–12^; left PT: *p* = 1.36). No significant difference was noted between GQI and QBI (right PT: *p* = 0.85; left PT: *p* = 0.44) or between DTI and DSI (right: *p* = 7.56 × 10^–4^; left PT: *p* = 1.03 × 10^–2^). The rankings of the number of true fibers obtained by the four diffusion models from greatest to least were as follows: GQI, QBI, DTI, and DSI (Figures 3A,B and Supplementary Table 1). The results of true fiber density were similar to the results of the number of true fibers (Figures 3E,F and Supplementary Tables 3, 4).

### Comparison of the Quantification of False Fibers Tracked Based on Four Diffusion Models

**FIGURE 4:**
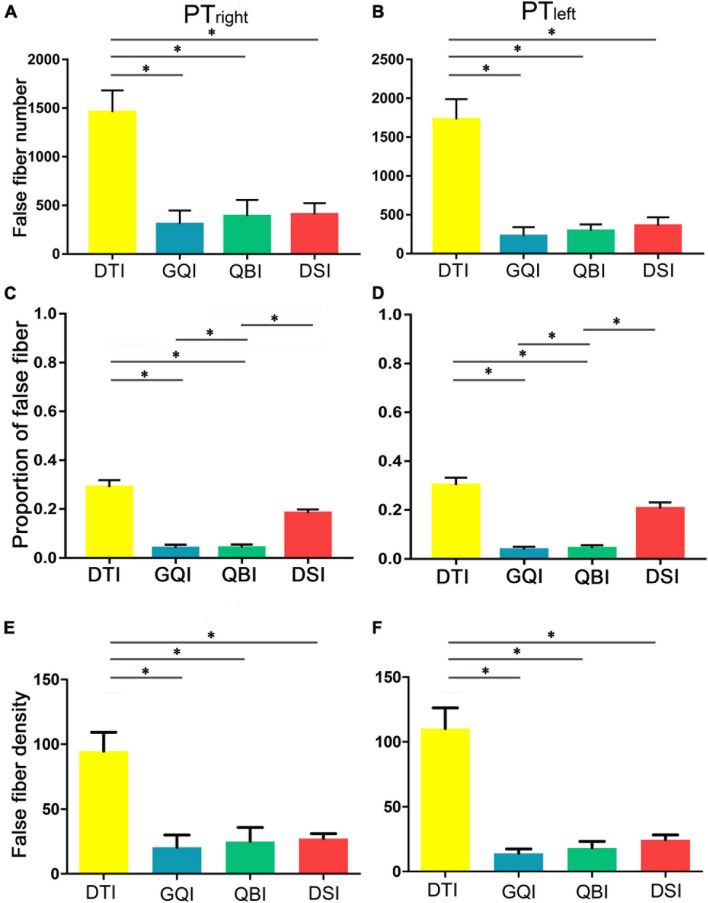
Comparison of the false fiber number and density based on four diffusion models. **(A)** The false fiber number of the right PT. **(B)** The false fiber number of the left PT. **(C)** The percentage of false fibers of the right PT. **(D)** The percentage of false fibers of the left PT. **(E)** The false fiber density of the right PT. **(F)** The false fiber density of the left PT. DTI, diffusion tensor imaging; DSI, diffusion spectral imaging; GQI, generalized Q-space sampling imaging; L, left; PT, pyramid tract; QBI, Q-ball imaging; R, right. * represents *p* < 0.0005 (0.05/96, Bonferroni corrected). The error bar represents standard errors.

Regarding the number of false fibers of bilateral PT (Figures 4A,B), one-way repeated-measure ANOVA revealed that there were statistically significant differences in the four diffusion models (right PT: *F* = 13.79, *p* = 5.10 × 10^–5^; left PT: *F* = 31.09, *p* = 4.97 × 10^–8^; Supplementary Table 5). Then, *post hoc* analysis was performed between any two diffusion models (Supplementary Table 6). The false fibers tracked based on the DTI diffusion model were the most common (Supplementary Table 5), and a significantly greater number of false fibers were tracked with the DTI compared with the GQI (right PT: *p* = 1.30 × 10^–4^; left PT: *p* = 1.21 × 10^–7^), QBI (right PT: *p* = 4.10 × 10^–4^; left PT: *p* = 3.35 × 10^–7^), and DSI models (right PT: *p* = 1.48 × 10^–5^; left PT: *p* = 1.44 × 10^–6^). No significant difference was noted among the QBI, GQI, and DSI (Supplementary Table 6). The rankings of the number of false fibers obtained by the four diffusion models from greatest to least were as follows: DTI, DSI, QBI, and GQI (Figures 4A,B and Supplementary Table 5). The results of the percentage of false fibers (the number of false fibers divided by the total number of fibers) were similar to the number of false fibers (Figures 4C,D and Supplementary Tables 5, 6), one-way repeated-measure ANOVA revealed statistically significant differences in the four diffusion models (right PT: *F* = 49.70, *p* = 6.05 × 10^–14^; left PT: *F* = 45.38, *p* = 3.11 × 10^–14^; Supplementary Table 5); the percentage of false fibers tracked based on the DTI diffusion model was significantly greater than those of GQI (right PT: *p* = 5.74 × 10^–10^; left PT: *p* = 1.97 × 10^–11^) and QBI (right PT: *p* = 1.79 × 10^–11^; left PT: *p* = 1.16 × 10^–11^). The percentage of false fibers tracked based on the DSI diffusion model was also significantly greater than those of GQI (right PT: *p* = 3.97 × 10^–9^; left PT: *p* = 1.77 × 10^–8^) and QBI (right PT = 4.36 × 45.12^–12^; left PT: *p* = 4.36 × 10^–8^). No significant difference was noted between the GQI and QBI (right PT: *p* = 0.85; left PT: *p* = 0.44), and no significant difference was noted between the DTI and DSI (right PT: *p* = 7.56 × 10^–4^; left PT: *p* = 1.03 × 10^–2^). The results of false fiber density were similar to the results of true fiber number (Figures 4E,F and Supplementary Tables 7, 8).

### Quantitative Comparison of the Number of True Fibers in Pyramidal Tracts Branches

**FIGURE 5:**
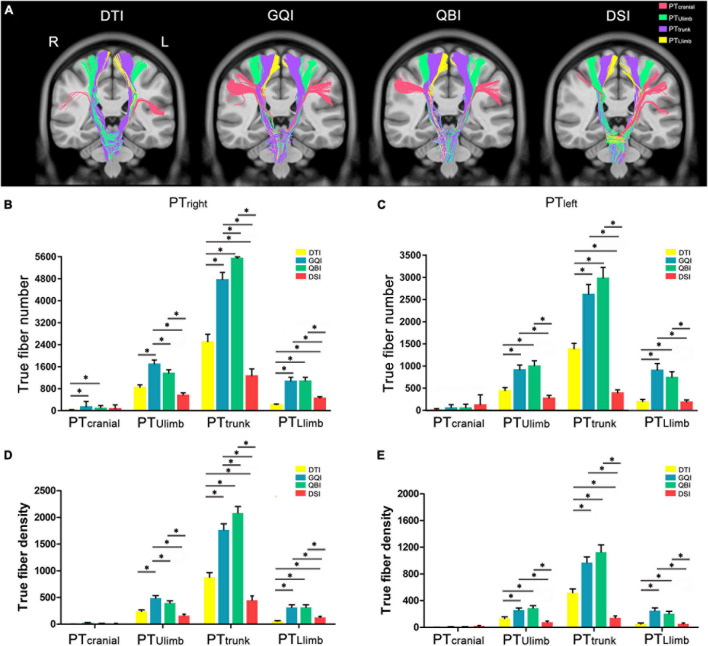
Comparison of the true fiber number and density in PT branches. **(A)** An example of the true fibers of PT branches tracked based on four diffusion models. **(B)** The true fiber number of the right PT branches. **(C)** The true fiber number of the left PT branches. **(D)** The true fiber density of the right PT branches. **(E)** The true fiber density of the left PT branches. DTI, diffusion tensor imaging; DSI, diffusion spectral imaging; GQI, generalized Q-space sampling imaging; L, left; PT, pyramid tract; QBI, Q-ball imaging; R, right. * represents *p* < 0.0005 (0.05/96, Bonferroni corrected). The error bar represents standard errors.

We further quantitatively compared the number of true fibers tracked based on four diffusion models in PT branches (Figure 5A). One-way repeated-measure ANOVA revealed statistically significant differences in the four diffusion models (except for the left PT_cranial_, all *p* < 0.003, Bonferroni correction; Supplementary Table 9). Then, *post hoc* analysis was performed between any two diffusion models (Supplementary Table 10). The results for PT_Ulimb_ and PT_trunk_ were similar to the number of true fibers of bilateral PT.

Regarding the right PT branches (Figure 5B), the number of PT_cranial_ tracked based on the DTI diffusion model was significantly less than those of GQI (*p* = 1.40 × 10^–4^) and QBI (*p* = 1.35 × 10^–4^), and the rankings of the number of true fibers in PT_cranial_ obtained by the four diffusion models from greatest to least were as follows: GQI, QBI, DSI, and DTI (Supplementary Table 9). The number of PT_Ulimb_ tracked based on the GQI diffusion model was significantly greater than those of DTI (*p* = 7.65 × 10^–7^), QBI (*p* = 3.78 × 10^–4^), and DSI (*p* = 4.51 × 10^–7^). The number of PT_Ulimb_ tracked based on the QBI diffusion model was significantly greater than that of DSI (*p* = 3.18 × 10^–5^). No significant difference was noted between DTI and QBI (*p* = 1.18 × 10^–3^) or between DTI and DSI (*p* = 6.28 × 10^–3^). The rankings of the number of true fibers of PT_Ulimb_ obtained by the four diffusion models from greatest to least were as follows: GQI, QBI, DTI, and DSI (Supplementary Table 9); the number of PT_*trunk*_ tracked based on the GQI diffusion model was significantly greater than that obtained with DTI (*p* = 7.24 × 10^–12^) and DSI (*p* = 6.49 × 10^–17^). The number of PT_*trunk*_ tracked based on the QBI diffusion model was significantly greater than that obtained with DTI (*p* = 7.09 × 10^–16^), GQI (*p* = 1.72 × 10^–8^), and DSI (*p* = 9.58 × 10^–20^). The number of PT_*trunk*_ tracked based on the DTI diffusion model was significantly greater than that obtained by DSI (*p* = 1.51 × 10^–9^). The rankings of the number of true fibers of PT_*trunk*_ obtained by the four diffusion models from greatest to least were as follows: QBI, GQI, DTI, and DSI (Supplementary Table 9). The number of PT_*Llimb*_ tracked based on the GQI diffusion model was significantly greater than those of DTI (*p* = 1.10 × 10^–7^) and DSI (*p* = 9.88 × 10^–5^). The number of PT_*Llimb*_ tracked based on the QBI diffusion model was significantly greater than those of DTI (*p* = 8.23 × 10^–6^) and DSI (*p* = 6.77 × 10^–5^). The number of PT_*Llimb*_ tracked based on the DSI diffusion model was significantly greater than that of DTI (*p* = 1.97 × 10^–6^). No significant differences were noted between GQI and QBI (*p* = 0.89). The rankings of the number of true fibers of PT_*Llimb*_ obtained by the four diffusion models from greatest to least were as follows: QBI, GQI, DSI, and DTI (Supplementary Table 9).

Regarding the left PT branches (Figure 5C), the number of true fibers in the PTcranial obtained based on the four diffusion models did not significantly differ. The rankings of the number of true fibers in PT_*cranial*_ obtained by the four diffusion models from greatest to least were as follows: DSI, QBI, GQI, and DTI (Supplementary Table 9). The number of PT_Ulimb_ tracked based on the GQI diffusion model was significantly greater than those of DTI (*p* = 3.35 × 10^–5^) and DSI (*p* = 6.02 × 10^–6^). The number of PT_Ulimb_ tracked based on the QBI diffusion model was significantly greater than those of DTI (*p* = 8.81 × 10^–7^) and DSI (*p* = 3.29 × 10^–6^). No significant differences were noted between any two groups (Supplementary Table 10), and the rankings of the number of true fibers in PT_Ulimb_ obtained by the four diffusion models from most to least were as follows: QBI, GQI, DTI, and DSI (Supplementary Table 9). The number of PT_*trunk*_ tracked based on the GQI diffusion model was significantly higher than those of DTI (*p* = 5.18 × 10^–7^) and DSI (*p* = 5.18 × 10^–13^). The number of PT_*trunk*_ tracked based on the QBI diffusion model was significantly greater than those of DTI (*p* = 9.06 × 10^–10^) and DSI (*p* = 2.88 × 10^–14^). The number of PT_*trunk*_ tracked based on the DTI diffusion model was significantly greater than that of DSI (*p* = 1.19 × 10^–10^). The rankings of the number of true fibers of PT_*trunk*_ obtained by the four diffusion models from greatest to least were as follows: QBI, GQI, DTI, and DSI. The number of PT_*Llimb*_ tracked based on the GQI diffusion model was significantly greater than those of DTI (*p* = 4.35 × 10^–6^) and DSI (*p* = 2.54 × 10^–5^). The number of PT_*Llimb*_ tracked based on the QBI diffusion model was significantly greater than those of DTI (*p* = 1.42 × 10^–5^) and DSI (*p* = 1.34 × 10^–4^). No significant differences were noted between any two groups (Supplementary Table 10). The rankings of the number of true fibers of PT_*Llimb*_ obtained by the four diffusion models from greatest to least were as follows: GQI, QBI, DTI, and DSI.

**FIGURE 6:**
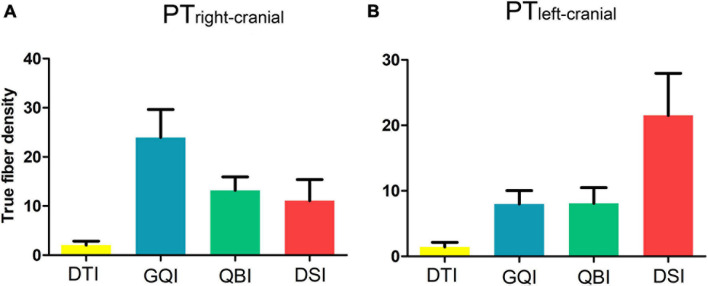
Comparison of the density of true fibers in PT_*cranial*_. **(A)** The true fiber density of the right PT_*cranial*_. **(B)** The true fiber density of the left PT_*cranial*_. DTI, diffusion tensor imaging; DSI, diffusion spectral imaging; GQI, generalized Q-space sampling imaging; L, left; PT, pyramid tract; QBI, Q-ball imaging; R, right. The error bar represents standard errors.

For both the left and right sides, more fibers of the PT_trunk_ were obtained based on each diffusion model compared with that of the PT_*cranial*_, PT_Ulimb_, and PT_Llimb_ branches. Furthermore, with the exception of PT_cranial_, the number of true fibers obtained based on GQI and QBI was also significantly greater than those obtained based on DTI and DSI (all *p* < 0.0005, Bonferroni corrected). The results of fiber density in PT_cranial_, PT_Ulimb_, and PT_Llimb_ were similar to the results of true fiber number (Figures 5D,E and Supplementary Tables 11, 12). The fiber density in the bilateral PTcranial obtained based on the four diffusion models did not significantly differ (right PT_cranial_: *F* = 6.48, *p* = 3.32 × 10 ^3^; left PT_cranial_: *F* = 5.65, *p* = 1.54 × 10 ^2^; Figures 6A,B and Supplementary Table 11).

**FIGURE 7:**
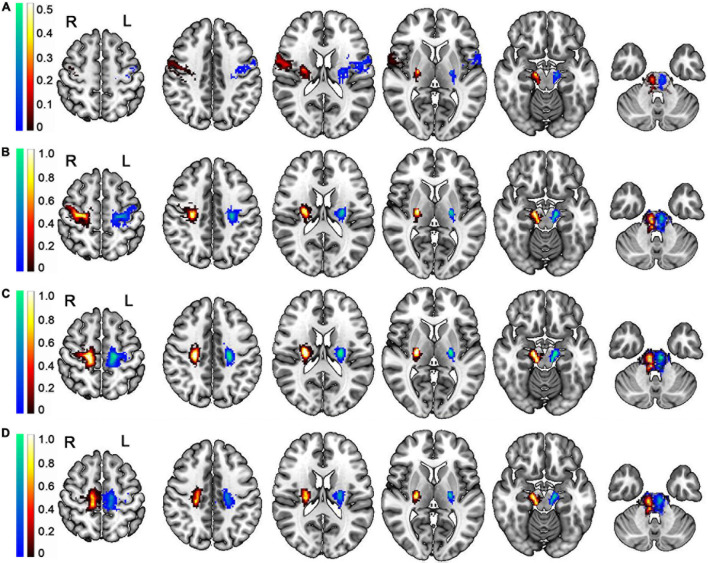
Group-wise probabilistic maps of the PT branches tracked based on the GQI diffusion model. **(A)** The group-wise probabilistic map of PT_ranial_. **(B)** The group-wise probabilistic map of PT_Ulimb_. **(C)** The group-wise probabilistic map of PT_trunk_. **(D)** The group-wise probabilistic map of PT_Llimb_. L, left; R, right; PT, pyramid tract; PT_cranial_, the head and face and tongue and larynx region; PT_Ulimb_, the hand region; PT_trunk_, the body region; PT_Llimb_, the foot region. The color bar represents the probability across participants; the warm color represents the probability of right PT branches, and the cold color represents the probability of left PT branches.

In addition, we produced groupwise probabilistic maps generated by averaging the normalized individual fibers of the PT branches based on the GQI diffusion model after comprehensively considering the true and false fibers (Figure 7), and the groupwise probabilistic maps are available at https://github.com/xinjunsuo/MR/issues/2. Meanwhile, we also provided the groupwise probabilistic maps generated by averaging the normalized individual fibers of the PT branches based on the DTI and QBI diffusion model (Supplementary Figures 1, 2). Compared with the GQI probability map, the left and right fibers in the brainstem position of all branches that produced the DTI probability map are basically indistinguishable.

## Discussion

Our present study aims to determine the best diffusion model for evaluating the quantification accuracy of PT and PT branches. Based on the high-quality HCP dataset, we found that GQI and QBI represented better diffusion models for PT and its branches compared with the DTI and DSI models. In addition, the fibers of PT_trunk_ obtained based on each diffusion model were much more abundant than those of PT_cranial_, PT_Ulimb_, and PT_Llimb_. Finally, we shared the groupwise probabilistic maps of the PT branches that were generated based on GQI using the HCP dataset in the present study. Our findings provide an objective reference for selecting diffusion models for PT and PT branches in clinical or scientific research. Furthermore, our findings contributed to the precisely locating the damaged regions based on the PT branches’ groupwise probabilistic maps.

Our study reveals the existence of more true fibers of the PT that was tracked based on the GQI and QBI diffusion models compared with the DTI and DSI models. The false fibers that reached the contralateral cerebral hemisphere were also tracked primarily based on the DTI and DSI diffusion models. The false fibers of the PT tracked based on the DTI diffusion model were the most common. After comprehensively considering the true and false fibers, the GQI diffusion model proves to be the best model for PT, and the DTI diffusion model the worst among the four diffusion models. These results are consistent with previous studies. Specifically, the GQI-based fiber tracking method achieved the highest valid connections (92%) among 96 methods in the ISMRM 2015 Tractography challenge (Wang et al., 2018), and the GQI diffusion model was superior to the DTI diffusion model in the display of nerve fibers of peritumoral edema (Zhang et al., 2013). Other scholars have suggested that QBI is more advantageous than DTI in spatial statistical and volumetric analysis of areas of interest (Corbo et al., 2014). The three diffusion models (DSI, QBI, and GQI) are non-parametric reconstruction models (Wedeen et al., 2005; Descoteaux et al., 2007; Yeh et al., 2010), and the advantage of this type of model is that the model assumptions do not limit them. These models do not assume a specific diffusion distribution structure. Thus, there is no risk of violating the model, and no overfitting problems are encountered in the model-based approach. In addition, these models do not require complex optimization or fitting compared with the DTI diffusion model and are less affected by outliers. Hence, the true fibers tracked based on these three models are better than the DTI model. The DSI diffusion model requires intensive sampling of q-space, which generally requires many different combinations of *q*-values and different directions (Wedeen et al., 2005; Glenn et al., 2016). Moreover, the data acquisition scheme of the HCP dataset only used three spherical sparse samplings rather than dense Cartesian q-space sampling. Therefore, the performance of the GQI and QBI was better than that of the DSI.

The Brainnetome atlas was used in our study as the reference for the division of M1, and the atlas was produced by conducting automatic tractography-based parcellation on the high-resolution imaging data provided by the HCP (Fan et al., 2016). Therefore, the atlas is undoubtedly the best for our study of the WM fibers of the PT and its branches. Moreover, for all PT branches on the left and right sides, the numbers of true fibers in each branch tracked by GQI and QBI were larger than those tracked by DTI and DSI. These findings also supported our recommendations for the GQI and QBI diffusion models. The fibers tracked based on each diffusion model were mainly concentrated in innervating the body area. In contrast, fewer fibers innervated the head and face and tongue and larynx areas. The number of fibers in the innervating upper limbs was larger than that in the lower limbs. More fibers indicate better information transfer, and the number of fibers was related to the difficulty of the action dominating the skeleton muscle (Liu et al., 2020; Pannek et al., 2020). Moreover, we shared the groupwise probabilistic maps of the PT branches based on the GQI diffusion model^5^ to provide more location information of each PT branch at the different sections for use in clinical practice and neuroscientific research and to supplement the gaps in research on the PT branch.

In addition, dMRI with multiple diffusion directions provided by the HCP dataset was used in our study. These data are recognized as high-quality data by many scholars for the quantitative evaluation of these four diffusion models. The anatomical travel path of the PT is relatively concentrated with minimal variation, and this information can be used as an objective reference to evaluate the advantages and disadvantages of different diffusion models.

Some limitations do exist in our study. First, due to the lack of precise measurements of motor ability, we could not establish a direct link between the PT branch and specific motor functions. Second, it is not clear which molecular and biological pathways modulate individual variance in the anatomical connectivity profiles of the PT and PT branches. Clarifying these issues will help to completely understand the functional roles of the PT and PT branches. Third, although we only used 50 subjects in HCP, our previous research has demonstrated the stability in HCP subjects (Suo et al., 2021). Finally, our comparison of the four diffusion models is mainly focused on demonstrating the performance of PT and PT branches rather than evaluating their computing time and other parameters.

## Conclusion

Our study provides a precise estimation of four diffusion models for the PT and PT branches. In scientific research on and clinical applications of the PT and PT branches, the GQI and QBI diffusion models are recommended among the four diffusion models (DTI, GQI, QBI, and GQI) after comprehensive assessment of quantitative indices. Moreover, we produced groupwise probability maps corresponding to each branch of the bilateral PT obtained based on the GQI diffusion model. We hope to provide a powerful reference for clinical treatment and research in related fields.

## Data Availability Statement

The datasets presented in this study can be found in online repositories. The names of the repository/repositories and accession number(s) can be found below: The group-wise probability maps generated by averaging the normalized individual fibers of the PT branches based on the GQI diffusion model are available at https://github.com/xinjunsuo/MR/issues/2. The HCP dataset that supports the findings of this study is available by Human Connectome Project at https://www.humanconnectome.org/study/hcp-young-adult.

## Ethics Statement

The human participants included in our study were from HCP Open Access Data. Ethical review and approval was not required for the current study in accordance with the local legislation and institutional requirements. Written informed consent for the current study was not required in accordance with the national legislation and the institutional requirements. The studies involving human participants in the analysed datasets followed the ethical standards of each institutional and/or national research committee and with the 1964 Helsinki declaration and its later amendments or comparable ethical standards, and written informed consent was obtained from all participants.

## Author Contributions

WQ: conceptualization, methodology, and writing—review and editing. XS: data curation and writing—original draft preparation. LG: visualization and methodology. DF: formal analysis. HD: software and validation. YL: data curation and validation. All authors contributed to the article and approved the submitted version.

## Conflict of Interest

The authors declare that the research was conducted in the absence of any commercial or financial relationships that could be construed as a potential conflict of interest.

## Publisher’s Note

All claims expressed in this article are solely those of the authors and do not necessarily represent those of their affiliated organizations, or those of the publisher, the editors and the reviewers. Any product that may be evaluated in this article, or claim that may be made by its manufacturer, is not guaranteed or endorsed by the publisher.

## Footnotes

1
http://protocols.humanconnectome.org/HCP/3T/imaging-protocols.html

2
https://fsl.fmrib.ox.ac.uk/fsl/fslwiki/FslInstallation

3
http://dsi-studio.labsolver.org

4
http://atlas.brainnetome.org

5
https://github.com/xinjunsuo/MR/issues/2
