# Supplementary material for: A Comparative Study of Diffusion Fiber Reconstruction Models for Pyramidal Tract Branches
Source: Front Neurosci. 2021 Dec 9;15:777377. doi: 10.3389/fnins.2021.777377 (PMC8698251; doi:10.3389/fnins.2021.777377)
Supplement: Supplementary file 1 [file Data_Sheet_1.docx]

A comparative study of diffusion fiber reconstruction models for pyramidal tract branches

Xinjun Suo^1, 2, 3, †^, Lining Guo^1, 2, †^, Dianxun Fu^1, 2^, Hao Ding^2, 3^, Yihong Li^3^, Wen Qin^1, 2, *^

^1^ Department of Radiology, and ^2^Tianjin Key Lab of Functional Imaging, Tianjin Medical University General Hospital, Tianjin 300052, China

^3^ School of Medical Imaging, Tianjin Medical University, Tianjin 300070, China

† These authors contribute equally to this work.

^*^ Correspondence: Wen Qin, Department of Radiology, and Tianjin Key Lab of Functional Imaging, Tianjin Medical University General Hospital. Anshan Road No. 154, Heping District, Tianjin 300052, China. Fax: +862260362260. Phone: +862260363760. Email: wayne.wenqin@gmail.com (W.Q.).

**Running title**: Diffusion models for pyramidal-tracts branches

## Supplementary Tables

**Supplementary Table 1: The ANOVA analysis of the quantification of true fibers tracked based on four diffusion models.**

|  | The true fiber number of the right PT | | The true fiber number of the left PT | | The percentage of true fibers of the right PT | | The percentage of true fibers of the left PT | |
| --- | --- | --- | --- | --- | --- | --- | --- | --- |
|  | Mean | SEM | Mean | SEM | Mean | SEM | Mean | SEM |
| DTI | 2704.64 | 217.03 | 2904.18 | 180.32 | 0.71 | 0.028 | 0.70 | 0.03 |
| GQI | 7280.66 | 356.17 | 6911 | 407.60 | 0.96 | 0.013 | 0.96 | 0.01 |
| QBI | 8360.22 | 1013.10 | 6867.7 | 414.85 | 0.96 | 0.013 | 0.96 | 0.01 |
| DSI | 2256.40 | 300.08 | 1875.9 | 299.16 | 0.82 | 0.015 | 0.79 | 0.02 |
| *F*-value | 33.01 | | 102.56 | | 49.70 | | 45.38 | |
| *P*-value | **6.81×10^-9^** | | **3.83×10^-26^** | | **6.05×10^-14^** | | **3.11×10^-14^** | |

Bold means the *P*-values could pass be corrected by Bonferroni correction (*P* < 0.003 (0.05/16)). Abbreviations: SEM = Standard Error of Mean.

**Supplementary Table 2: The *P*-values of post-hoc analysis performed on the quantification of true fibers tracked based on four diffusion models.**

|  | The true fiber number of the right PT | The true fiber number of the left PT | The percentage of true fibers of the right PT | The percentage of true fibers of the left PT |
| --- | --- | --- | --- | --- |
| DTI vs GQI | **8.38×10^-19^** | **6.67×10^-14^** | **5.74×10^-10^** | **1.97×10^-11^** |
| DTI vs QBI | **1.09×10^-6^** | **4.78×10^-14^** | **1.79×10^-11^** | **1.16×10^-11^** |
| DTI vs DSI | 2.17×10^-1^ | 5.99×10^-3^ | 7.56×10^-4^ | 1.03×10^-2^ |
| GQI vs QBI | 2.80×10^-1^ | 8.17×10^-1^ | 8.45×10^-1^ | 4.42×10^-1^ |
| GQI vs DSI | **1.05×10^-14^** | **3.47×10^-16^** | **3.97×10^-9^** | **1.77×10^-8^** |
| QBI vs DSI | **3.66×10^-7^** | **4.92×10^-16^** | **6.45×10^-12^** | **4.36×10^-8^** |

Bold means the *P*-values could pass be corrected by Bonferroni correction (*P* < 0.0005 (0.05/96)).

**Supplementary Table 3: The ANOVA analysis of the quantification of true fiber density tracked based on four diffusion models.**

|  | The true fiber density of the right PT | | The true fiber density of the left PT | |
| --- | --- | --- | --- | --- |
|  | Mean | SEM | Mean | SEM |
| DTI | 172.44 | 13.14 | 189.11 | 12.82 |
| GQI | 473.56 | 26.11 | 447.92 | 29.07 |
| QBI | 555.18 | 77.40 | 445.16 | 29.81 |
| DSI | 148.90 | 19.71 | 121.76 | 18.63 |
| *F*-value | 26.72 | | 92.014 | |
| *P*-value | **2.65×10^-7^** | | **2.81×10^-23^** | |

Bold means the *P*-values could pass be corrected by Bonferroni correction (*P* < 0.003 (0.05/16)).

**Supplementary Table 4: The *P*-values of post-hoc analysis performed on the quantification of true fiber density tracked based on four diffusion models.**

|  | The true fiber density of the right PT | The true fiber density of the left PT |
| --- | --- | --- |
| DTI vs GQI | **4.42×10^-17^** | **6.76×10^-13^** |
| DTI vs QBI | **8.53×10^-6^** | **7.12×10^-13^** |
| DTI vs DSI | 2.95×10^-1^ | 4.30×10^-3^ |
| GQI vs QBI | 2.73×10^-1^ | 8.32×10^-1^ |
| GQI vs DSI | **1.06×10^-14^** | **3.37×10^-15^** |
| QBI vs DSI | **3.27×10^-6^** | **4.81×10^-15^** |

Bold means the *P*-values could pass be corrected by Bonferroni correction (*P* < 0.0005 (0.05/96)).

**Supplementary Table 5: The ANOVA analysis of the quantification of false fibers tracked based on four diffusion models.**

|  | The false fiber number of the right PT | | The false fiber number of the left PT | | The percentage of false fibers of the right PT | | The percentage of false fibers of the left PT | |
| --- | --- | --- | --- | --- | --- | --- | --- | --- |
|  | Mean | SEM | Mean | SEM | Mean | SEM | Mean | SEM |
| DTI | 1459.56 | 223.14 | 1729.16 | 259.08 | 0.29 | 0.03 | 0.30 | 0.03 |
| GQI | 307.26 | 140.72 | 226.1 | 56.86 | 0.04 | 0.01 | 0.04 | 0.01 |
| QBI | 389.12 | 166.97 | 292.88 | 83.94 | 0.04 | 0.01 | 0.04 | 0.01 |
| DSI | 407.58 | 57.54 | 359.08 | 53.57 | 0.18 | 0.02 | 0.21 | 0.02 |
| *F*-value | 13.79 | | 31.09 | | 49.70 | | 45.38 | |
| *P*-value | **5.10×10^-5^** | | **4.97×10^-8^** | | **6.05×10^-14^** | | **3.11×10^-14^** | |

Bold means the *P*-values could pass be corrected by Bonferroni correction (*P* < 0.003 (0.05/16)). Abbreviations: SEM = Standard Error of Mean.

**Supplementary Table 6: The *P*-values of post-hoc analysis performed on the quantification of false fibers tracked based on four diffusion models.**

|  | The false fiber number of the right PT | The false fiber number of the left PT | The percentage of false fibers of the right PT | The percentage of false fibers of the left PT |
| --- | --- | --- | --- | --- |
| DTI vs GQI | **1.30×10^-4^** | **1.21×10^-7^** | **5.74×10^-10^** | **1.97×10^-11^** |
| DTI vs QBI | **4.10×10^-4^** | **3.35×10^-7^** | **1.79×10^-11^** | **1.16×10^-11^** |
| DTI vs DSI | **1.48×10^-5^** | **1.44×10^-6^** | 7.56×10^-4^ | 1.03×10^-2^ |
| GQI vs QBI | 1.72×10^-1^ | 3.48×10^-1^ | 8.45×10^-1^ | 4.42×10^-1^ |
| GQI vs DSI | 5.00×10^-1^ | 6.73×10^-2^ | **3.97×10^-9^** | **1.77×10^-8^** |
| QBI vs DSI | 9.17×10^-1^ | 4.90×10^-1^ | **6.45×10^-12^** | **4.36×10^-8^** |

Bold means the *P*-values could pass be corrected by Bonferroni correction (*P* < 0.0005 (0.05/96)).

**Supplementary Table 7: The ANOVA analysis of the quantification of false fiber density tracked based on four diffusion models.**

|  | The false fiber density of the right PT | | The false fiber density of the left PT | |
| --- | --- | --- | --- | --- |
|  | Mean | SEM | Mean | SEM |
| DTI | 94.51 | 14.71 | 110.18 | 16.01 |
| GQI | 20.58 | 9.47 | 14.11 | 3.31 |
| QBI | 25.00 | 10.97 | 18.21 | 5.04 |
| DSI | 27.34 | 3.87 | 24.46 | 3.87 |
| *F*-value | 13.17 | | 32.06 | |
| *P*-value | **7.70×10^-5^** | | **3.06×10^-8^** | |

Bold means the *P*-values could pass be corrected by Bonferroni correction (*P* < 0.003 (0.05/16)).

**Supplementary Table 8: The *P*-values of post-hoc analysis performed on the quantification of false fiber density tracked based on four diffusion models.**

|  | The false fiber density of the right PT | The false fiber density of the left PT |
| --- | --- | --- |
| DTI vs GQI | **1.79×10^-4^** | **1.22×10^-7^** |
| DTI vs QBI | **4.78×10^-4^** | **3.36×10^-7^** |
| DTI vs DSI | **2.79×10^-5^** | **6.17×10^-7^** |
| GQI vs QBI | 1.70×10^-1^ | 3.80×10^-1^ |
| GQI vs DSI | 4.96×10^-1^ | 2.56×10^-2^ |
| QBI vs DSI | 8.41×10^-1^ | 3.02×10^-1^ |

Bold means the *P*-values could pass be corrected by Bonferroni correction (*P* < 0.0005 (0.05/96)).

**Supplementary Table 9: The ANOVA analysis of the number of true fibers in PT branches tracked based on four diffusion models.**

|  | Right PT_cranial_ | | Right PT_Ulimb_ | | Right PT_trunk_ | | Right PT_Llimb_ | | Left PT_cranial_ | | Left PT_Ulimb_ | | Left PT_trunk_ | | Left PT_Llimb_ | | |
| --- | --- | --- | --- | --- | --- | --- | --- | --- | --- | --- | --- | --- | --- | --- | --- | --- | --- |
|  | Mean | SEM | Mean | SEM | Mean | SEM | Mean | SEM | Mean | SEM | Mean | SEM | Mean | SEM | Mean | SEM |  |
| DTI | 10.78 | 4.05 | 837.26 | 99.49 | 2489.58 | 284.63 | 198.82 | 38.82 | 8.26 | 4.10 | 436.56 | 79.09 | 1387.90 | 135.13 | 190.90 | 57.47 |  |
| GQI | 128.56 | 28.99 | 1682.62 | 161.53 | 4740.36 | 275.08 | 1055.42 | 154.31 | 44.92 | 11.70 | 907.32 | 116.11 | 2625.74 | 232.81 | 897.08 | 158.22 |  |
| QBI | 75.10 | 15.70 | 1350.38 | 137.35 | 5513.48 | 275.16 | 1062.34 | 147.44 | 44.88 | 13.21 | 992.98 | 127.55 | 2994.24 | 251.82 | 736.48 | 13527 |  |
| DSI | 59.50 | 21.69 | 551.26 | 97.27 | 1251.66 | 264.02 | 439.08 | 66.42 | 115.72 | 33.16 | 267.38 | 71.72 | 389.54 | 76.73 | 181.98 | 54.11 |  |
| *F*-value | 7.12 | | 23.70 | | 141.23 | | 27.33 | | 5.91 | | 22.10 | | 74.08 | | 21.09 | | |
| *P*-value | **1.77×10^-3^** | | **7.62×10^-8^** | | **9.41×10^-25^** | | **3.86×10^-7^** | | 1.24×10^-2^ | | **3.16×10^-8^** | | **2.74×10^-16^** | | **6.00×10^-6^** | | |

Bold means the *P*-values could pass be corrected by Bonferroni correction (*P* < 0.003 (0.05/16)). Abbreviations: SEM = Standard Error of Mean.

**Supplementary Table 10: The *P*-values of post-hoc analysis performed on the number of true fibers in PT branches tracked based on four diffusion models.**

|  | Right PT_cranial_ | Right PT_Ulimb_ | Right PT_trunk_ | Right PT_Llimb_ | Left PT_cranial_ | Left PT_Ulimb_ | Left PT_trunk_ | Left PT_Llimb_ |
| --- | --- | --- | --- | --- | --- | --- | --- | --- |
| DTI vs GQI | **1.40×10^-4^** | **7.65×10^-7^** | **7.24×10^-12^** | **1.10×10^-7^** | 1.04×10^-3^ | **3.35×10^-5^** | **5.18×10^-7^** | **4.35×10^-6^** |
| DTI vs QBI | **1.35×10^-4^** | 1.18×10^-3^ | **7.09×10^-16^** | **8.23×10^-8^** | 7.19×10^-3^ | **8.81×10^-7^** | **9.06×10^-10^** | **1.42×10^-5^** |
| DTI vs DSI | 2.92×10^-2^ | 6.28×10^-3^ | **1.51×10^-9^** | **1.97×10^-6^** | 2.23×10^-3^ | 8.45×10^-2^ | **1.19×10^-10^** | 7.96×10^-1^ |
| GQI vs QBI | 1.40×10^-2^ | **3.78×10^-4^** | **1.72×10^-8^** | 8.90×10^-1^ | 9.97×10^-1^ | 1.45×10^-1^ | 5.89×10^-4^ | 1.02×10^-2^ |
| GQI vs DSI | 6.08×10^-2^ | **4.51×10^-7^** | **6.49×10^-17^** | **9.88×10^-5^** | 5.04×10^-2^ | **6.02×10^-6^** | **5.18×10^-13^** | **2.54×10^-5^** |
| QBI vs DSI | 5.61×10^-1^ | **3.18×10^-5^** | **9.58×10^-20^** | **6.77×10^-5^** | 6.23×10^-2^ | **3.29×10^-6^** | **2.88×10^-14^** | **1.34×10^-4^** |

Bold means the *P*-values could pass be corrected by Bonferroni correction (*P* < 0.0005 (0.05/96)).

**Supplementary Table 11: The ANOVA analysis of the true fiber density in PT branches tracked based on four diffusion models.**

|  | Right PT_cranial_ | | Right PT_Ulimb_ | | Right PT_trunk_ | | Right PT_Llimb_ | | Left PT_cranial_ | | Left PT_Ulimb_ | | Left PT_trunk_ | | Left PT_Llimb_ | |
| --- | --- | --- | --- | --- | --- | --- | --- | --- | --- | --- | --- | --- | --- | --- | --- | --- |
|  | Mean | SEM | Mean | SEM | Mean | SEM | Mean | SEM | Mean | SEM | Mean | SEM | Mean | SEM | Mean | SEM |
| DTI | 86.68 | 54.87 | 10.87 | 1.43 | 0.71 | 132.13 | 24.98 | 518.38 | 58.30 | 51.41 | 15.26 | 86.68 | 54.87 | 10.87 | 1.43 | 0.71 |
| GQI | 112.53 | 311.40 | 51.65 | 7.99 | 2.04 | 259.18 | 32.24 | 969.47 | 87.96 | 249.54 | 42.77 | 112.53 | 311.40 | 51.65 | 7.99 | 2.04 |
| QBI | 122.88 | 313.08 | 50.09 | 8.08 | 2.41 | 287.75 | 37.81 | 1127.80 | 109.10 | 204.54 | 37.17 | 122.88 | 313.08 | 50.09 | 8.08 | 2.41 |
| DSI | 82.75 | 126.63 | 19.10 | 21.51 | 6.44 | 75.51 | 19.47 | 142.09 | 29.10 | 51.22 | 14.79 | 82.75 | 126.63 | 19.10 | 21.51 | 6.44 |
| *F*-value | 6.48 | | 23.22 | | 109.72 | | 20.99 | | 5.65 | | 22.20 | | 62.53 | | 22.08 | |
| *P*-value | 3.32×10^-3^ | | **1.73×10^-7^** | | **5.52×10^-19^** | | **8.00×10^-6^** | | 1.54×10^-2^ | | **1.91×10^-8^** | | **3.58×10^-15^** | | **4.00×10^-6^** | |

Bold means the *P*-values could pass be corrected by Bonferroni correction (*P* < 0.003 (0.05/16)).

**Supplementary Table 12: The *P*-values of post-hoc analysis performed on the true fiber density in PT branches tracked based on four diffusion models.**

|  | Right PT_cranial_ | Right PT_Ulimb_ | Right PT_trunk_ | Right PT_Llimb_ | Left PT_cranial_ | Left PT_Ulimb_ | Left PT_trunk_ | Left PT_Llimb_ |
| --- | --- | --- | --- | --- | --- | --- | --- | --- |
| DTI vs GQI | **2.88×10^-4^** | **1.20×10^-6^** | **3.41×10^-10^** | **2.38×10^-6^** | 8.22×10^-4^ | **3.71×10^-5^** | **9.03×10^-7^** | **2.04×10^-6^** |
| DTI vs QBI | **1.27×10^-4^** | **9.55×10^-4^** | **2.15×10^-13^** | **2.18×10^-6^** | 8.09×10^-3^ | **1.33×10^-6^** | **1.93×10^-8^** | **1.09×10^-5^** |
| DTI vs DSI | 3.98×10^-2^ | 5.13×10^-3^ | **1.89×10^-10^** | **2.24×10^-6^** | 3.17×10^-3^ | 5.87×10^-2^ | **2.10×10^-9^** | 9.86×10^-1^ |
| GQI vs QBI | 1.19×10^-2^ | 6.60×10^-4^ | **1.14×10^-7^** | 9.16×10^-1^ | 9.60×10^-1^ | 9.07×10^-2^ | 1.74×10^-3^ | 6.98×10^-3^ |
| GQI vs DSI | 7.58×10^-2^ | **5.26×10^-7^** | **4.60×10^-15^** | **4.44×10^-4^** | 5.25×10^-2^ | **3.83×10^-6^** | **1.07×10^-12^** | **1.61×10^-5^** |
| QBI vs DSI | 6.83×10^-1^ | **4.51×10^-5^** | **3.27×10^-17^** | **3.76×10^-4^** | 6.64×10^-2^ | **2.54×10^-6^** | **1.80×10^-12^** | **1.32×10^-4^** |

Bold means the *P*-values could pass be corrected by Bonferroni correction (*P* < 0.0005 (0.05/96)).

**Supplementary Figures**

**
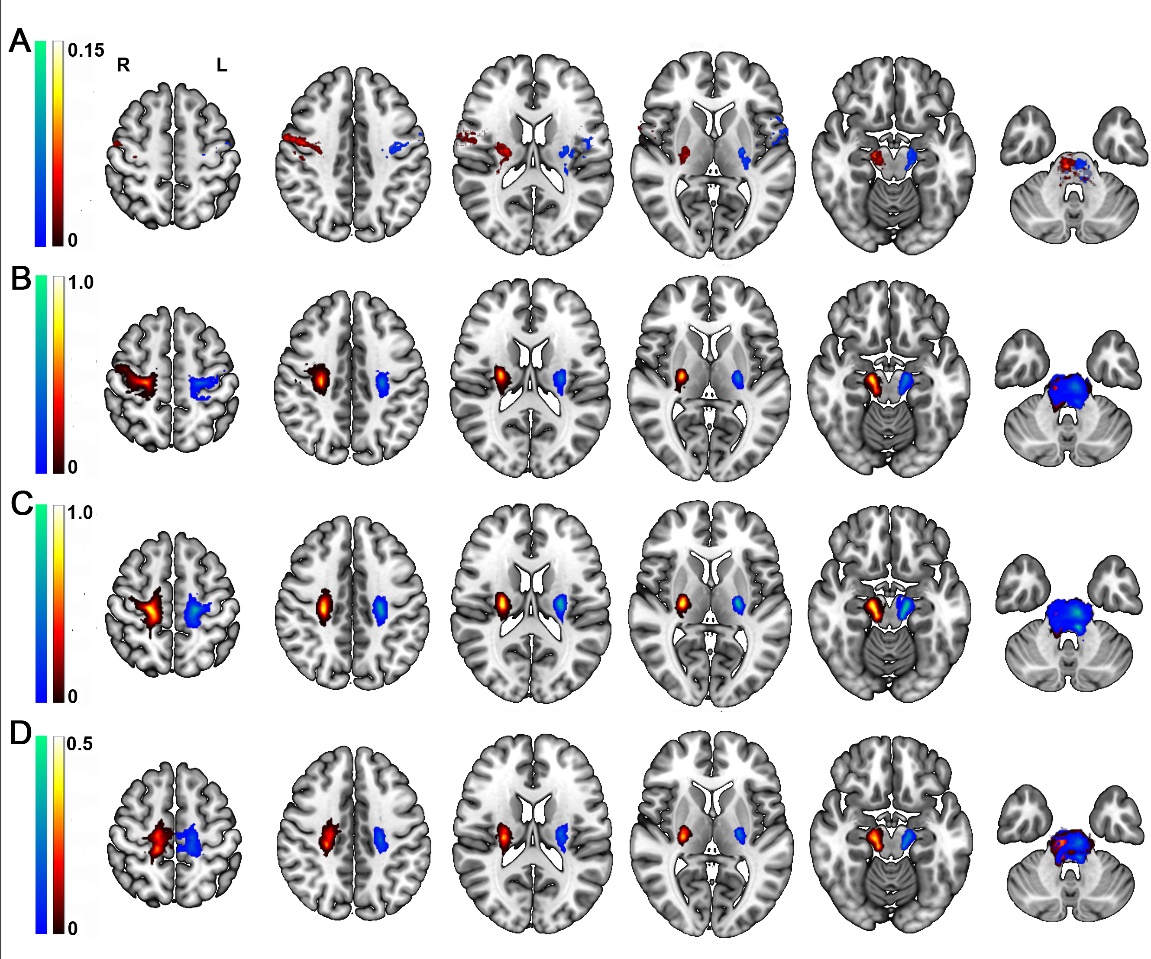
**

**Figure 1. Group-wise probabilistic maps of the PT branches Group-wise probabilistic maps of the PT branches tracked based on DTI diffusion model.** (**A**) The group-wise probabilistic map of PT_cranial_. (**B**) The group-wise probabilistic map of PT_Ulimb_. (**C**) The group-wise probabilistic map of PT_trunk_. (**D**) The group-wise probabilistic map of PT_Llimb_. Abbreviations: L = left, R = right, PT = pyramid tract, PT_cranial_ = the head and face and tongue and larynx region, PT_Ulimb_ = the hand region, PT_trunk_ = the body region, PT_Llimb_ = the foot region. The color bar represents the probability across participants, the cold color represents the probability of left PT branches, the warm color represents the probability of right PT branches.


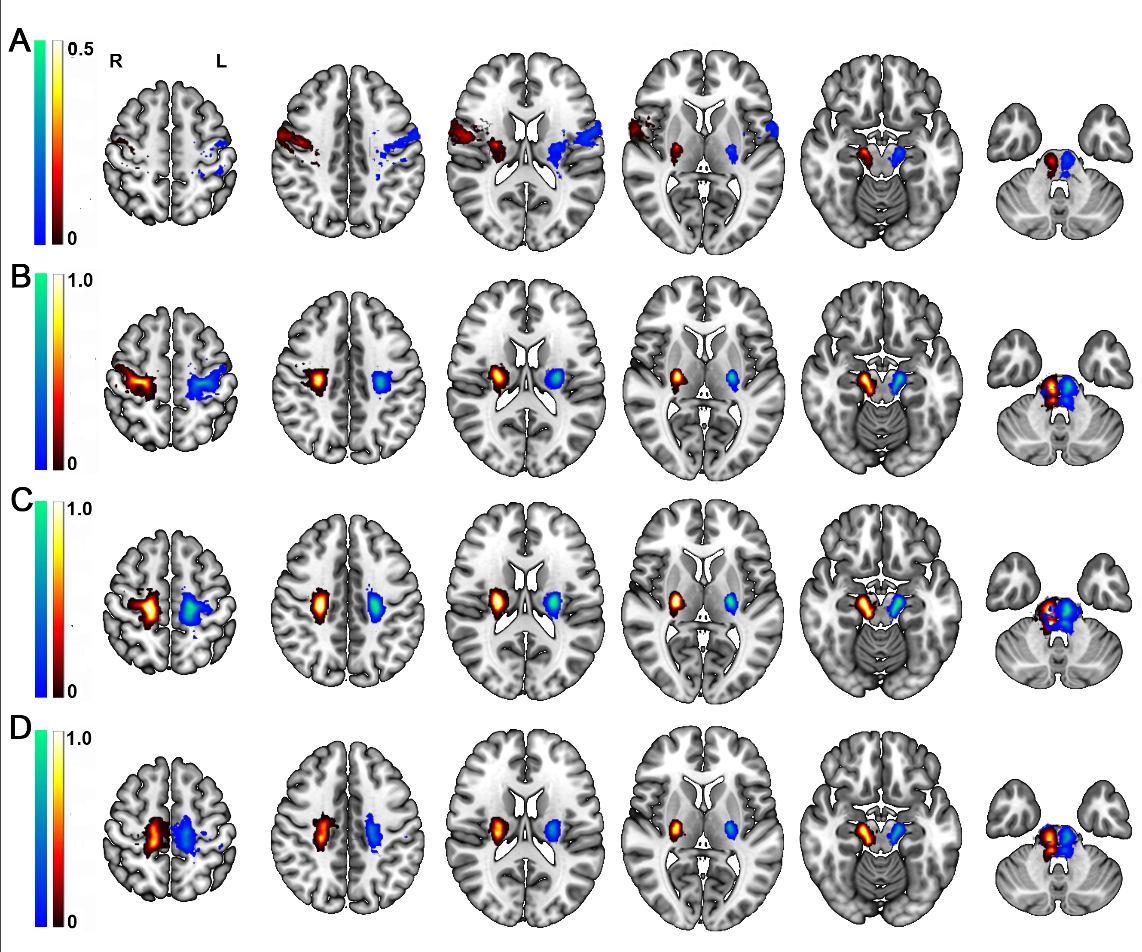


**Figure 2. Group-wise probabilistic maps of the PT branches Group-wise probabilistic maps of the PT branches tracked based on QBI diffusion model.** (**A**) The group-wise probabilistic map of PT_cranial_. (**B**) The group-wise probabilistic map of PT_Ulimb_. (**C**) The group-wise probabilistic map of PT_trunk_. (**D**) The group-wise probabilistic map of PT_Llimb_. Abbreviations: L = left, R = right, PT = pyramid tract, PT_cranial_ = the head and face and tongue and larynx region, PT_Ulimb_ = the hand region, PT_trunk_ = the body region, PT_Llimb_ = the foot region. The color bar represents the probability across participants, the cold color represents the probability of left PT branches, the warm color represents the probability of right PT branches.
